# Olfactory learning without the mushroom bodies: Spiking neural network models of the honeybee lateral antennal lobe tract reveal its capacities in odour memory tasks of varied complexities

**DOI:** 10.1371/journal.pcbi.1005551

**Published:** 2017-06-22

**Authors:** HaDi MaBouDi, Hideaki Shimazaki, Martin Giurfa, Lars Chittka

**Affiliations:** 1 School of Biological and Chemical Sciences, Queen Mary University of London, London, United Kingdom; 2 RIKEN Brain Science Institute, Saitama, Japan; 3 Research Centre on Animal Cognition, Center for Integrative Biology, CNRS, University of Toulouse, Toulouse, France; CHB, Harvard Medical School, UNITED STATES

## Abstract

The honeybee olfactory system is a well-established model for understanding functional mechanisms of learning and memory. Olfactory stimuli are first processed in the antennal lobe, and then transferred to the mushroom body and lateral horn through dual pathways termed medial and lateral antennal lobe tracts (m-ALT and l-ALT). Recent studies reported that honeybees can perform elemental learning by associating an odour with a reward signal even after lesions in m-ALT or blocking the mushroom bodies. To test the hypothesis that the lateral pathway (l-ALT) is sufficient for elemental learning, we modelled local computation within glomeruli in antennal lobes with axons of projection neurons connecting to a decision neuron (LHN) in the lateral horn. We show that inhibitory spike-timing dependent plasticity (modelling non-associative plasticity by exposure to different stimuli) in the synapses from local neurons to projection neurons decorrelates the projection neurons’ outputs. The strength of the decorrelations is regulated by global inhibitory feedback within antennal lobes to the projection neurons. By additionally modelling octopaminergic modification of synaptic plasticity among local neurons in the antennal lobes and projection neurons to LHN connections, the model can discriminate and generalize olfactory stimuli. Although positive patterning can be accounted for by the l-ALT model, negative patterning requires further processing and mushroom body circuits. Thus, our model explains several–but not all–types of associative olfactory learning and generalization by a few neural layers of odour processing in the l-ALT. As an outcome of the combination between non-associative and associative learning, the modelling approach allows us to link changes in structural organization of honeybees' antennal lobes with their behavioural performances over the course of their life.

## Author summary

The honeybee olfactory system offers the opportunity to study different levels of learning complexity in a small size network. Odour information is transferred from the antennae to the antennal lobes, and from there to the mushroom bodies and the lateral horn via parallel medial and lateral tracts of projection neurons. Although much progress has been made in understanding olfactory coding in the bee brain, the precise contribution of the lateral pathway in olfactory learning is still unclear. To understand the computational mechanisms underpinning the lateral antennal lobe tract, we modelled local computation and non-associative plasticity within glomeruli in the antennal lobe and the lateral horn where they were additionally modulated by octopamine to achieve associative learning. We establish that the connectivity within the antennal lobe (that is shaped by non-associative plasticity) can also be modified by inhibitory feedback neurons. This makes output patterns of antennal lobe more separable and sparser. Our modelling indicates that bees, using the lateral pathway, might learn to solve positive patterning tasks in addition to elemental learning and olfactory generalisation without the contribution of the mushroom bodies. Yet, the model cannot account for negative patterning, thus indicating that the mushroom bodies may be required for this discrimination. Our modelling approach allows us to link changes in structural organization of honeybees' antennal lobes with behavioural performance over the course of their life.

## Introduction

Olfactory coding and its modification by learning have been extensively studied in the honeybee, *Apis mellifera*, both at the behavioural and neural levels [1–5]. Honeybees are able to discriminate between odours, or mixtures of odours, and generalise from a trained odour to perceptually similar odours [6–10]. The protocol typically used to study these capacities is the olfactory conditioning of the proboscis extension reflex (PER) [3,11,12]. The protocol relies on pairing an odorant as a conditioned stimulus with sucrose solution as a reward signal, i.e. as an unconditioned stimulus; in this case, the bee learns to associate the conditioned stimulus with the reward and subsequently responds with proboscis extension to the conditioned stimulus [13,14]. In certain forms of non-elemental olfactory learning (configural learning), bees are trained to discriminate single odorants from their mixture; reinforcement assigned to the single odours has a different valence compared to that of the odour mixture, so that ambiguity arises at the level of odour components [6,15,16]. For instance, in negative patterning discrimination, two individual odours A and B are rewarded while the mixture AB is non-rewarded (i.e. A+, B+ vs. AB-). During training, each odour component is as often rewarded as non-rewarded so that discrimination requires learning for instance that A alone is different from A in the presence of B. Interestingly, honeybees learn to solve negative patterning discriminations [6] while fruit flies *Drosophila melanogaster* are unable to learn this task [6,17,18]. However, the key circuitries underlying this cognitive capacity have only recently started to be elucidated [5,19].

**Fig 1:**
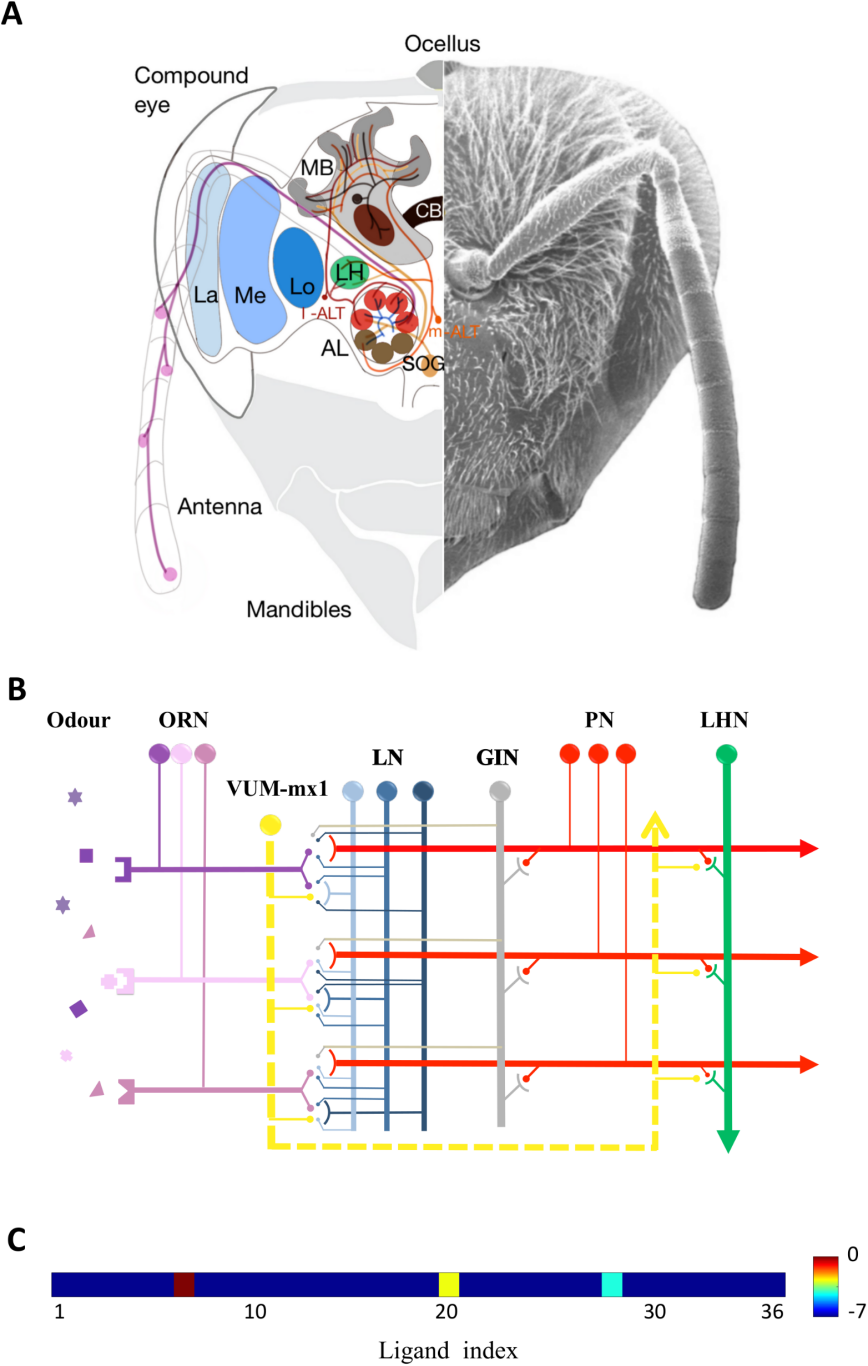
Schematic view of the honeybee olfactory system. (A) Frontal view of morphological connectivity of olfactory pathways. The antennal lobe is the primary site of olfactory processing which receives input from ~60,000 olfactory receptor neurons (ORNs) distributed along the placode sensilla on the antenna. ORNs project to 65 glomeruli that contain 800 excitatory projection neurons (PNs) and 4000 inhibitory local neurons (LNs). Two distinct groups of glomeruli within the antennal lobe (AL) are shown in brown and red spheres specialized for m-PNs and l-PNs. Glomeruli are laterally interconnected by a set of local inhibitory neurons (blue neurons). Axonal PNs extend from the antennal lobe to higher processing centres, such as the mushroom bodies (MB) and Lateral horn (LH) via two tracts, the medial antennal lobe tract (m-ALT, brown) and the lateral antennal lobe tract (l-ALT, red). An octopaminergic neuron (in yellow), VUM-mx1 projects from suboesophageal ganglion (SOG) to three areas of honeybee brain, AL, MB calyces and LH which represents reinforcement signal. Electron micrograph by Axel Brockmann [98]; figure design by Marie Guiraud. (B) The model network of the honeybee lateral antennal lobe tract. The model uses 36 ORNs types (in pink) that are activated by odorants (shown by different shapes; squares, triangles and stars). One ORN responds to multiple ligands of odorants with different sensitivities (One ligand can activate multiple ORNs). ORNs of the same type (i.e., the same sensitivity to ligands) project to PNs and LNs in the same glomerulus. Inhibitory LNs interact with PNs and LNs, both in other glomeruli. More specifically, PNs are disinhibited by the LN-LN connections. Although each glomerulus includes dendrites of several PNs, only one PN and LN are shown for the 3 glomeruli. PNs send axons into LH for connection with a single decision neuron, LHN. The VUM-mx1 neuron modulates inhibitory spike timing-dependent plasticity (iSTDP) of LN-LN and PN-LHN synapses. (C) An artificial odorant stimulus shown as a vector representation. Elements of the vector represent concentration of 36 different ligands. A single odour was modelled by a vector consisting of 2 to 5 active elements because an odour typically contains 2 to 5 ligands. Here, the concentration of each ligand (log[C]) in the odour vector was displayed in colours ranging from blue (lowest concentration) to red (highest concentration).

A honeybee’s antennae contain ~60,000 olfactory receptor neurons that transform chemical features of the environment into spatiotemporal patterns of neural activity (Fig 1) [20]. Axons of different types of olfactory receptor neurons extend to a primary olfactory centre, the antennal lobe that contains 165 spherical structures known as glomeruli (Fig 1A) [21,22]. Glomeruli are sites of synaptic contacts between afferents of olfactory receptors, inhibitory local neurons (LNs) connecting glomeruli, and excitatory projection neurons (PNs) (~800) conveying the processed olfactory message to higher-order centres such as mushroom bodies and the lateral horn. Two types of inhibitory local neurons, heterogeneous and homogeneous, are distinguished in the antennal lobe, depending on their arborisation pattern [21–24]. The glomeruli are laterally interconnected via local neurons or indirectly through projection neurons (Fig 1A and 1B) [4,22,24]. Most projection neurons convey odour information to higher brain regions through a dual pathway [25,26]. Projection neurons located in the dorsal region of the antennal lobe form the so-called lateral antennal lobe tract (l-ALT) which extends to the lateral horn and then further to the mushroom bodies. Projection neurons in the ventral region of the antennal lobe form the medial antennal lobe tract (m-ALT), which first projects to the mushroom bodies and then to the lateral horn (for more detail see; [4,27–29]). Interestingly, l-ALT projection neurons can be found in honeybees and other Hymenoptera but not in *Drosophila*, which only has m-ALT projection neurons in its olfactory system [28,30]. Recent studies suggested that different features of odorants might be processed separately by these two parallel tracts of projection neurons [25,26]. Sucrose reward representation is mediated by a giant octopaminergic neuron termed the VUMmx1 neuron, whose activity can substitute for real sucrose in PER olfactory conditioning [31]. Importantly, VUMmx1 contacts the olfactory circuit at three main regions, the antennal lobes, the mushroom bodies, and the lateral horn, thus providing multiple, spatially segregated opportunities for odour-sucrose associations. Although much progress has been made in understanding the physiological properties of projection neurons belonging to these pathways, the roles of these parallel pathways and their contribution to elemental and non-elemental olfactory learning is still unknown.

Computational models have been developed to understand functions and mechanisms of these pathways. A recent firing-rate model of the antennal lobe demonstrated that inhibitory local neurons with a global gain neuron could replicate different coding characteristics of the l-ALT and m-ALT pathways [32]. It also has been shown that lateral inhibition provided by local interneurons increases linear separability of odour representations in the antennal lobes, and improves the linear classifier in odour discrimination [33]. The m-ALT pathway that feeds into the mushroom bodies is thought to play a central role in olfactory learning and memory. Heisenberg’s model [34], which is followed by most computational models of associative learning, describes how odour information is encoded in the Kenyon cells (the mushroom bodies’ constitutive neurons) and the connections that the m-ALT projection neurons make with them. Wessnitzer et al. [19] modelled the *Drosophila* olfactory system from the neural coding stage in the antennal lobe to the mushroom body extrinsic neurons. Their spiking neural network can learn both elemental and non-elemental conditioning tasks, similarly to a recent model of the honeybee mushroom body [5]. Earlier studies produced ambiguous results referring to the question of whether bees can learn elemental associations without the higher-order processing provided by the mushroom bodies [35,36]. However, a recent study used selective pharmacological blocking of mushroom bodies and of sub-areas of these structures, and showed that in the absence of functional mushroom bodies, bees fail at learning complex (configural) discrimination but can still learn simple olfactory discrimination [15].

Although computational models have focused on the mushroom bodies in analyses of associative olfactory learning, it is usually neglected that both the antennal lobe and the lateral horns possess the basic circuitry to support olfactory learning (i.e. connectivity between odour and sucrose pathways) [4,29]. Hence, we here explore the potential olfactory learning capacities of the l-ALT, i.e. the circuit from olfactory receptor neurons to the lateral horn via the antennal lobes, using a neural network model.

We first focus on olfactory receptor models and modelled their responses to a panel of different odorants. The model reproduces realistic patterns of neural activity at the input level of the antennal lobes. We then implement a non-associative learning rule in the synaptic connections of local neurons to projection neurons of the antennal lobe. We show that exposing the model to different stimuli results in a rearrangement of the initial random inhibitory lateral connections, which then form a local connectivity pattern within the antennal lobes. This promotes separation of odour representations in the antennal lobes. Next, we incorporate VUMmx1 signalling and enrich our model with octopamine-modulated plasticity in the antennal lobes and the lateral horn to model associative olfactory learning (i.e. learning of odour-sucrose associations). We compare the model output with behavioural data from different learning paradigms including elemental learning, configural discriminations, and olfactory generalization. We find that the neural circuit of the l-ALT model accounts for elemental learning and positive patterning discrimination, but not for negative patterning discrimination. In addition, the model can generalize a learned positive patterning discrimination to novel stimuli. The inability of our model to solve the negative patterning confirms the experimental finding that the mushroom bodies are necessary for some forms of configural learning. The model also supports the asymmetric nature of generalization between certain pairs of odorants reported for bees.

## Results

### Characteristics of olfactory receptor neurons

Odours are detected by olfactory receptor neurons, which are located within specialized structures called sensilla, distributed on the surface of the antennae. Axons of olfactory receptor neurons constitute the antennal nerve that project to the antennal lobe and provide odour information to this first olfactory processing centre. Since olfactory receptor neurons have selective but also overlapping odour-response-profiles [37], an odour may activate more than one type of olfactory receptor. The odour-response profiles are modelled and described by using Eq 3 in the Methods Section, which allows generating dose-response curves. Each olfactory receptor neuron exhibits unique response curves, and saturates at a different ligand concentration [38]. These diversities are represented by a matrix of receptor affinity (See Method and S1 Fig) that controls the sensitivity of olfactory receptor neurons to different concentration levels (S2 Fig) [30,39]. We used a fixed affinity matrix throughout this study.

**Fig 2:**
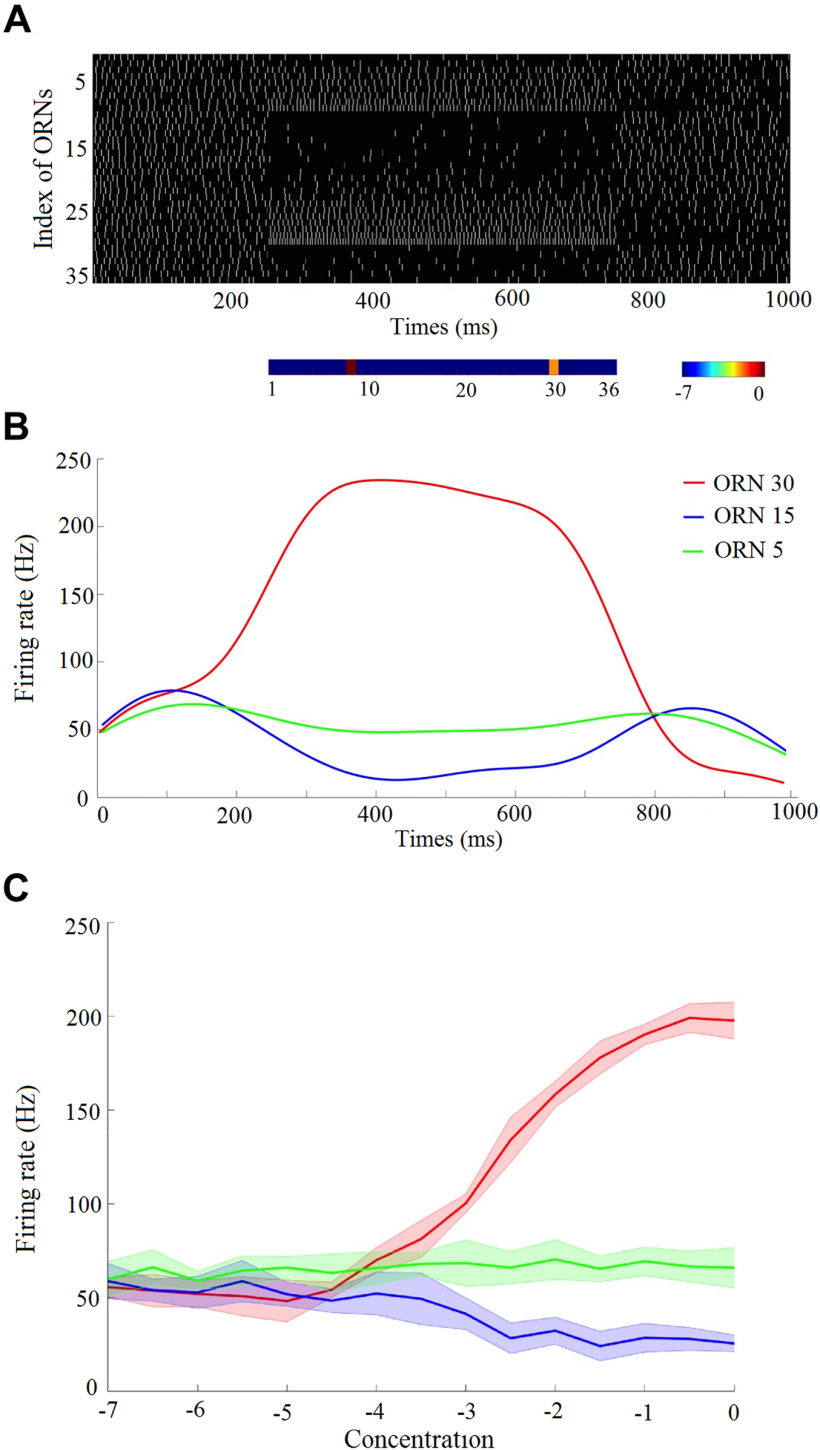
Firing rate properties of the stimulated olfactory receptor neurons. A) Simulated spontaneous and evoked spiking activity of a group of 36 olfactory receptor neuron (ORN) types for 1000 ms. The raster plot exhibits high spontaneous activity before and after the evoked activity of a stimulus (shown below with two active ligands) at times 250 ms and 750 ms. Multiple ORN types are activated by a single ligand. B) Firing rates of 3 different ORNs evoked by the odorant. These exemplary firing rates show a same odour stimulus excites (red) or inhibits (blue) olfactory receptor neurons, and some receptors are insensitive to the odour (green). ORN responses are dynamic and those sensitive ORNs fire most strongly at the stimuli onset. C) Mean and standard error (SE) of the firing rates of three different ORNs across 50 trials are plotted as a function of the ligand concentration which. Blue and red curves show how ligands of an odour suppress and activate the receptor's spike rate below and above the spontaneous activity. Error bars = SE.

We simulated spontaneous and evoked spiking activity of 36 types of olfactory receptor neurons during 1000 ms (Fig 2). The evoked activity was induced by an odour stimulus presented 250 ms after the onset of this period and which lasted 500 ms (i.e. until 750 ms). Simulation continued during further 250 ms to complete the 1000 ms. The olfactory receptor neurons exhibit high spontaneous activity rate (Fig 2A), which in turn maintains the high activity of projection neurons in the absence of stimuli. This allows a single projection neuron to code different odours at different concentrations by increasing or decreasing its firing rate from the spontaneous rate [38]. Multiple types of olfactory receptor neurons are activated by a single ligand. Fig 2B shows exemplary firing rates of three olfactory receptor neurons that are activated by the same input stimulus. The olfactory receptor neurons quickly respond to the olfactory stimulation and return to baseline activity after removing the stimulus [30]. Increasing odour concentration increases or decreases their responses from baseline level (Fig 2C). Modelling responses of olfactory receptor neurons in this way reproduces the variable selectivity and sensitivity of real olfactory receptor neurons with different tuning responses (S3 Fig) [40].

### Non-associative learning in the antennal lobe

**Fig 3:**
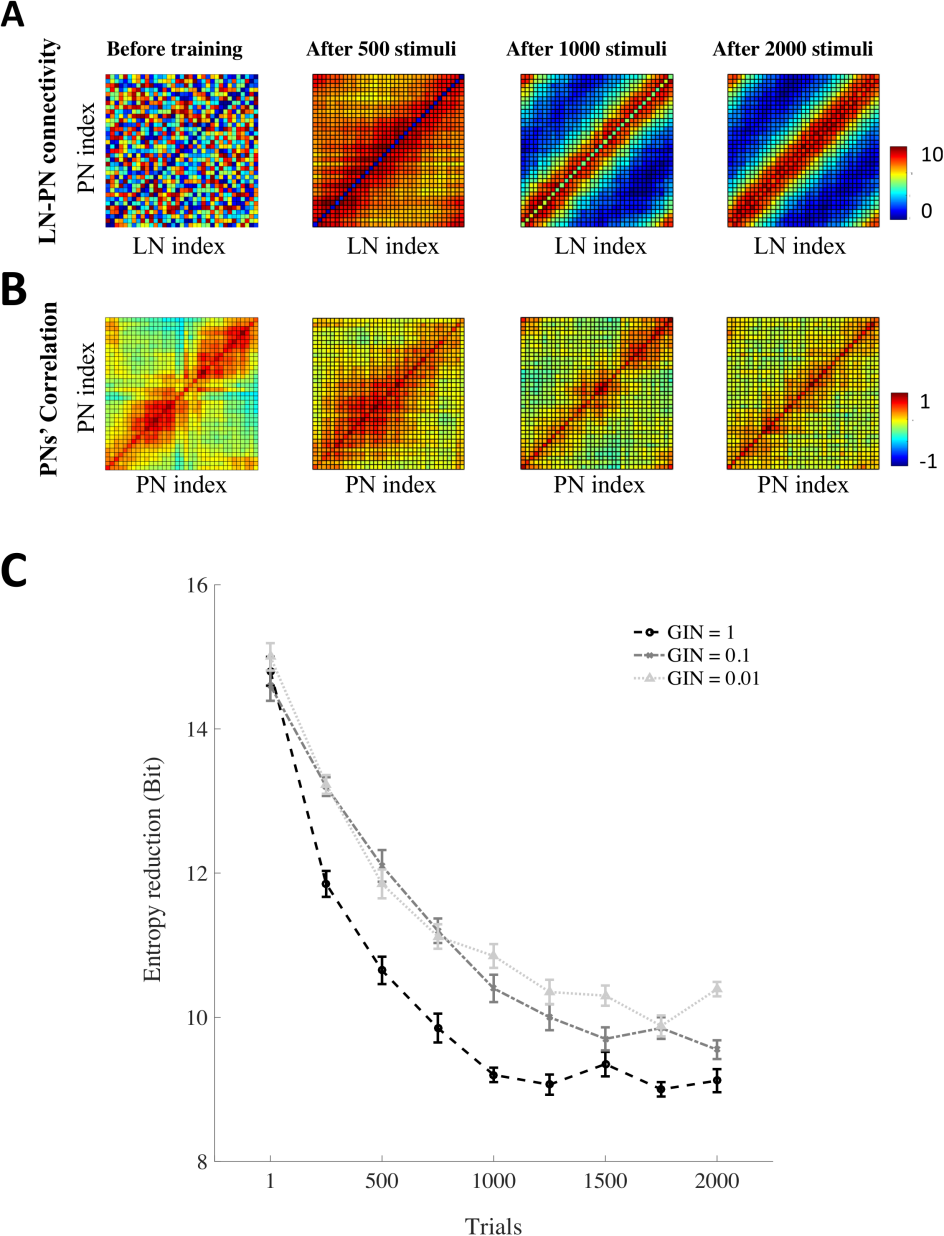
Non-associative plasticity in the antennal lobe and the effect of inhibitory feedback on network decorrelation. (A) Weight matrices of the synaptic connectivity from 36 antennal lobe local neurons to 36 projection neurons (PNs) in the presence of iSTDP between these connections (From left to right: random weights before training; weights after 500, 1000 and 2000 stimuli presentations). Each column of matrices exhibits strength of an inhibitory antennal lobe local neuron connection to different PNs. The initial connectivity matrix (left) was generated by a random Gaussian distribution, *N*(0, 10); (see S1 Video). (B) Correlation matrices of PN outputs before and after the exposure to stimuli. Positive and negative correlations are coloured by red and blue respectively. The correlation matrices approaches to a diagonal matrix, indicating that PN activity becomes decorrelated over training. Correlation matrices are calculated from the PNs’ firing rate activated by 64 different stimuli. This comparison shows that correlations between PNs are reduced over different stimulus presentation. C) The entropy reduction that measures the strength of correlations between PNs is plotted as a function of the number of presented odour. The entropy reductions of the PNs' activity of 20 different simulated bees (different initial conditions and a different set of 2000 stimuli) are plotted as a function of the number of stimuli presentation for different values of the global inhibitory neuron (GIN) (Black for strong inhibitory feedback and grey for weak inhibitory feedback). Here low entropy reduction indicates less correlation. The entropy reduces after more odours are presented to the model. Increasing the inhibitory feedback signal from GIN accelerates decorrelation of PN activity.

The functional units of the antennal lobes are the glomeruli, where different types of neurons converge and connect to each other. Each glomerulus is made of synaptic contacts between excitatory afferent axons of olfactory receptor neurons, inhibitory local neurons, and excitatory projection neurons conveying the reshaped olfactory message to higher order centres. It has been shown that non-associative learning (synaptic plasticity in the absence of reward, i.e. upon odour exposure) changes neural activity in the antennal lobes [41]. Here, we modelled such non-associative learning by a symmetric inhibitory spike timing-dependent plasticity (iSTDP) in synaptic connections of local neurons to projection neurons. We then expose the antennal lobe model to a sequence of random odours in the presence of this iSTDP. Fig 3A illustrates weight matrices of the synaptic connectivity from 36 local neurons to 36 projection neurons throughout the simulation. Each matrix column shows the strength of an inhibitory local neuron connection to projection neurons. The initial random matrix is reformed to a structured local connectivity matrix between the glomeruli, which represents local connectivity within the antennal lobe (see S1 Video) [24,42]. Thus, the inputs to projection neurons are modified according to the state of activity across the antennal lobe. As a result, the correlation of projection neurons’ output approaches an uncorrelated diagonal matrix (Fig 3B). This means that the activity of projection neurons is decorrelated as a result of non-associative learning by exposing the glomeruli to different stimuli [43]. In order to assess the decorrelation process of projection neurons, we quantified correlations by reduction of the entropy of projection neurons’ activity from their independent activity. This Entropy reduction was calculated by $ER=\frac{1}{2}\;\log\left({\left(2\pi e\right)}^{36}|\Sigma |\right)-\frac{1}{2}\;\log\left({\left(2\pi e\right)}^{36}\right)$, where |∑| is the determinant of covariance matrix, ∑, obtained from activities of 36 projection neurons (Fig 3C). In addition, we examined the contribution of global inhibitory feedback neurons by changing their synaptic strength. Importantly, we found that the strength of global inhibitory neuron (homogeneous local neuron) regulates the redundancy reduction processing in the antennal lobes (Fig 3C) [32,44]. Thus, the structured lateral inhibition existing in the antennal lobe improves the capacity of linear detectors in the next layer to extract pattern identity [45].

### Effect of the homogeneous local inhibitory neuron on odour separation and sparseness in the antennal lobe

**Fig 4:**
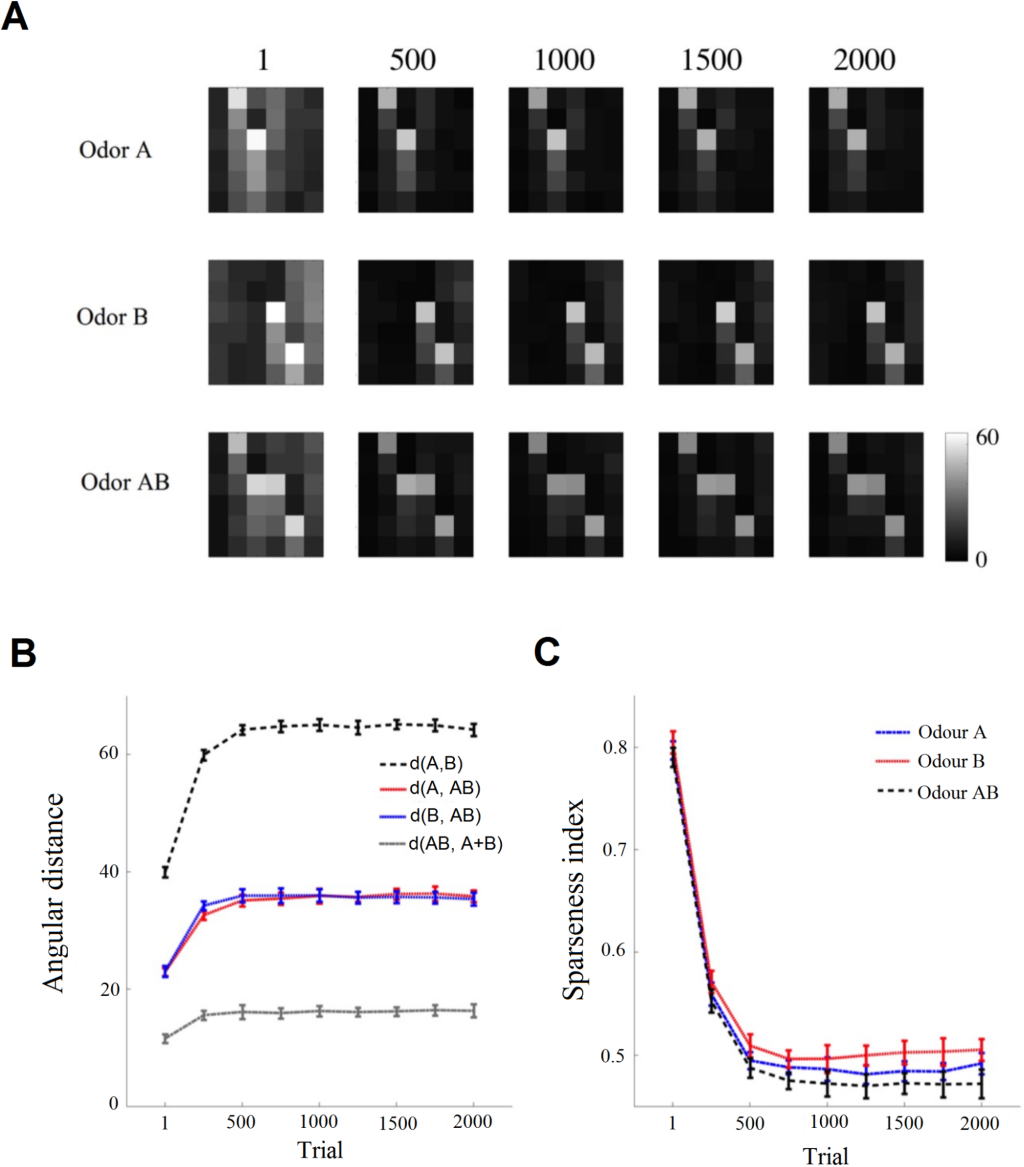
Example of pattern activity of dorsal glomeruli output (response of olfactory projection neurons). A) Different odorants cause different activation patterns in the dorsal region of the antennal lobes (AL). Each row of matrices exhibits the antennal lobe activity through the non-associative learning for three different odours (A, B and the odour mixture AB). Matrices show the odour representation of PNs in the dorsal region of AL containing 36 projection neurons (PNs). They are arranged in a square with 6 × 6 pixels. The colour of elements (i, j) shows a firing rate of *PN*_i*j_. B) Angular distance between PN responses for odour A, odour B, or odour AB are plotted for 50 different simulated bees (mean+- SE). The structured inhibitory connectivity from antennal lobe local neurons to PNs enhances separation between activity patterns for stimuli in the antennal lobe. C) Average activity sparseness for odour representations in the antennal lobes during the training. The low sparseness index corresponds to high sparseness population activity.

We investigated the separation of odorant representations arising in the antennal lobes as a consequence of non-associative learning. To study the effect of inhibitory neurons within the antennal lobe on the output of projection neurons, an angular distance between two vectors, *P*_1_, *P*_2_ that display the population activity of projection neurons for two odours was calculated by $d=arccos(\frac{P_{1}.P_{2}}{\left|P_{1}||P_{2}\right|})$, where ‘.’ indicates the inner product between two vectors *P*_1_, *P*_2_, and | *x* | represents magnitude of the vector *x*. By measuring the angular distance between the activities of projection neurons in the antennal lobe for odours A and B, we found that the neural representation of the two different odours was more separated by exposure to different odorants (Fig 4B). Here, we tracked an angular distance between the population activities of projection neurons across glomeruli for stimulus A, stimulus B and their mixture AB. (Fig 4B). Odours A and B activate two different but overlapping sets of glomeruli (neural representations of odours A and B in the antennal lobes). Given the proposed connectivity between local and projection neurons, presenting odours A and B together activates some of the projection neurons within glomeruli corresponding to both odours and activates a new set of projection neurons that were silent in presenting odours A or B. The new activated projection neurons constitute the neural response corresponding to the interaction between odours A and B. At strong activity of the global inhibitory neuron, the lateral inhibitory network pushes activity down and enhances the inter-glomerular contrast. This arrangement is compatible with observed data from the honeybee antennal lobes [46,47]. Moreover, strong inhibition across glomeruli has been reported for odour mixtures [47,48].

To compare the population sparseness within the antennal lobes during the simulation, we used the Treves-Rolls measures [49] of sparseness index, $SI=(\sum_{j=1}^{36}r_{j}/36{)}^{2}/(\sum_{j=1}^{36}{r_{j}}^{2}/36)$, where *r*_*j*_ is the firing-rate of the *j*th projection neuron. We then compared the sparseness index of antennal lobes for an odour mixture AB with those corresponding to stimuli A or B alone. Fig 4C predicts the role of lateral inhibition in sparse coding, as demonstrated in sensory processing of various modalities [50]. This shows that the activity pattern of projection neurons gets sparser (and the contrast of neural representation is enhanced) when the global inhibitory neuron becomes stronger. It also indicates that fewer neurons are activated when the olfactory system experiences more odours, which is more energy efficient [51]. This result predicts that the sparse representation of odours in different regions of the antennal lobe might be adjusted by different distributions of inhibitory signals.

Taken together, our assumptions on non-associative plasticity and local inhibition in the antennal lobe network can reduce the correlation between the responses of projection neurons and reproduce their tuning responses to different stimuli, both for m-ALT and l-ALT projection neurons. Since we were interested in the contributions of the l-ALT to different forms of olfactory learning and generalization, we defined a connectivity matrix between local interneurons and projection neurons with a strong inhibitory component and studied the capacity of this matrix to account for associative olfactory learning.

### Olfactory conditioning task

We tested the performance of our model of the l-ALT pathway in a set of different learning paradigms. To this end, the network model was enriched with octopamine modulation of synaptic plasticity in the antennal lobe and lateral horn, consistent with octopamine-based signalling of sucrose reward in these regions via the VUMmx1 neuron (Fig 1B, see Introduction).

**Fig 5:**
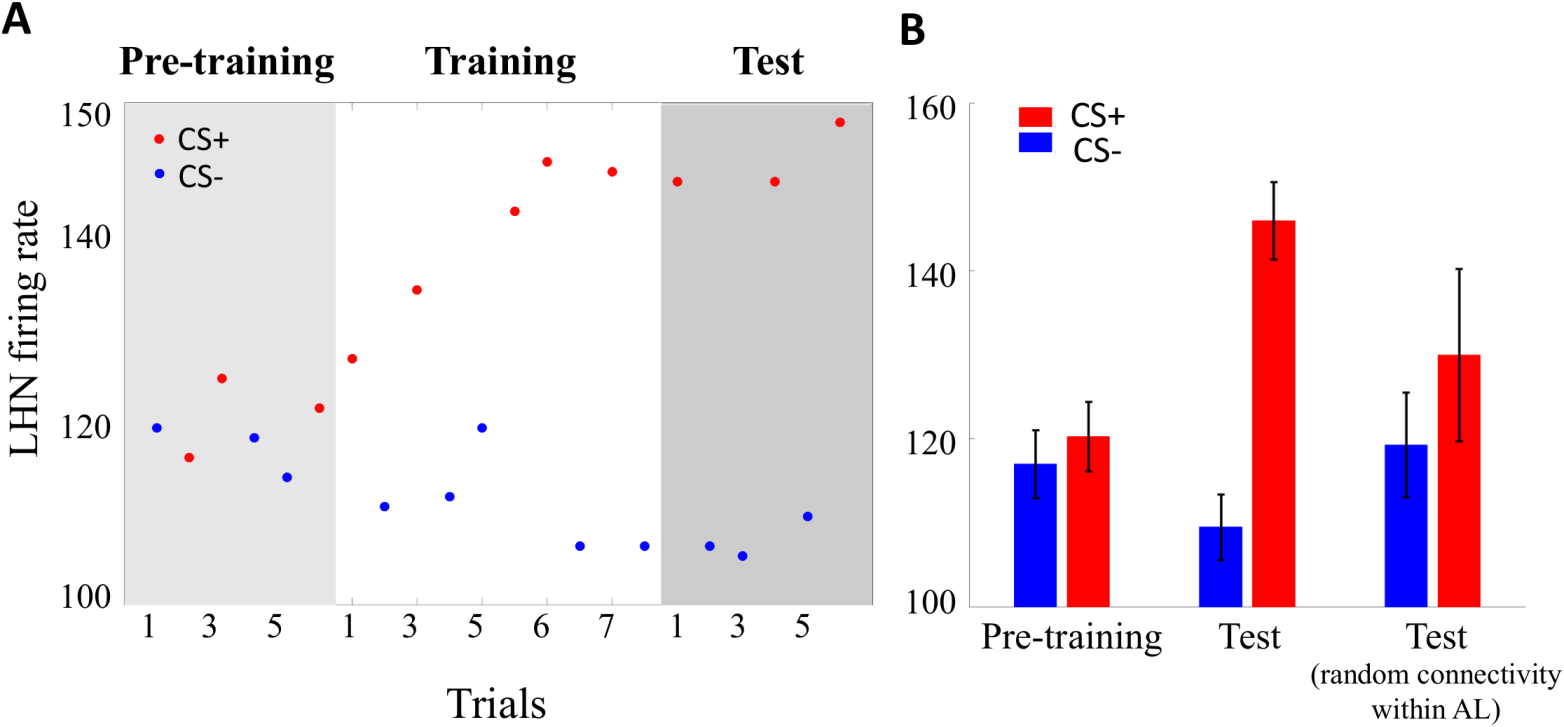
Model performance in differential olfactory conditioning of the proboscis extension reflex. A) Firing rates of the LHN response to a rewarded odour A (CS+) and unrewarded odour B (CS-) during three stages of the PER task; pre-training, training, and test. The red and blue points show responses of the LHN to the CS+ and CS-, respectively. Synaptic strengths between antennal lobe projection neurons to the LHN are modified only during the training (white area). Conditioning the model with CS+ induces increased firing rate in LHN during training. B) Responses of LHN to both CS+ and CS- before and after the conditioning for two different models, one with the structured connectivity and the other with random connectivity within the antennal lobe. The red and blue bars represent the LHN activity for CS+ and CS-, respectively. Standard error (SE) bars were calculated from the LHN’s firing rate for 50 different odours and different initial parameters in the differential conditioning. Bees were able to learn to discriminate significantly between rewarded stimuli and unrewarded stimuli (p-value < 10^−6^) while bees with random connectivity between local neurons and projection neurons cannot distinguish the CS+ (p-value = 0.29).

We first trained the model using a differential conditioning task, an elemental form of learning in which an odorant A is paired with sucrose reward (CS+) during the stimulus presentation for 500 ms while another odorant B is delivered without reward (CS-). Bees trained in this way easily learn the discrimination and extend the proboscis to A and not to B. Since the lateral horn is thought to be a premotor area [52–54], the strong response of the lateral horn neuron to odour A would translate into proboscis extension response to this odorant. Hence, we assumed that the lateral horn neuron (LHN) acts as a decision neuron, showing a stronger response for CS+ than to CS-. After training, performance of the model is measured by an average firing rate of the lateral horn neuron obtained from presenting odour A and B (for 3 times randomly) without reinforcement. Fig 5A provides an example of the differential conditioning task, and shows that the firing rate of the lateral horn neuron increases after presentation of the CS+ and tends to decrease after CS- delivery. This figure implies that the maximum difference between responses of the lateral horn neuron to CS+ and CS- are obtained after only three presentation of CS+. In order to replicate the learning task for different bees and different odours, we repeated the simulation, using different initial parameters and different odours. The firing rate of lateral horn neuron for the CS+ was significantly higher than that for the CS- (p-value < 10^−6^) while there was no difference between them before training (Fig 5B). This indicates that the model is able to reproduce the elemental discrimination learning underlying differential olfactory conditioning [3]. Conversely, the model with fixed random connectivity between local neurons and projection neurons within the antennal lobe cannot discriminate between positive and negative conditional stimuli (p-value = 0.29). A comparison between the results of these models reveals how the proper inhibitory connectivity between neurons in the antennal lobe can enhance the learning performance of the model. This emphasizes the importance of the structured connectivity that emerges in the antennal lobe by the non-associative learning in the performance of bees in olfactory learning tasks.

### Olfactory generalization

**Fig 6:**
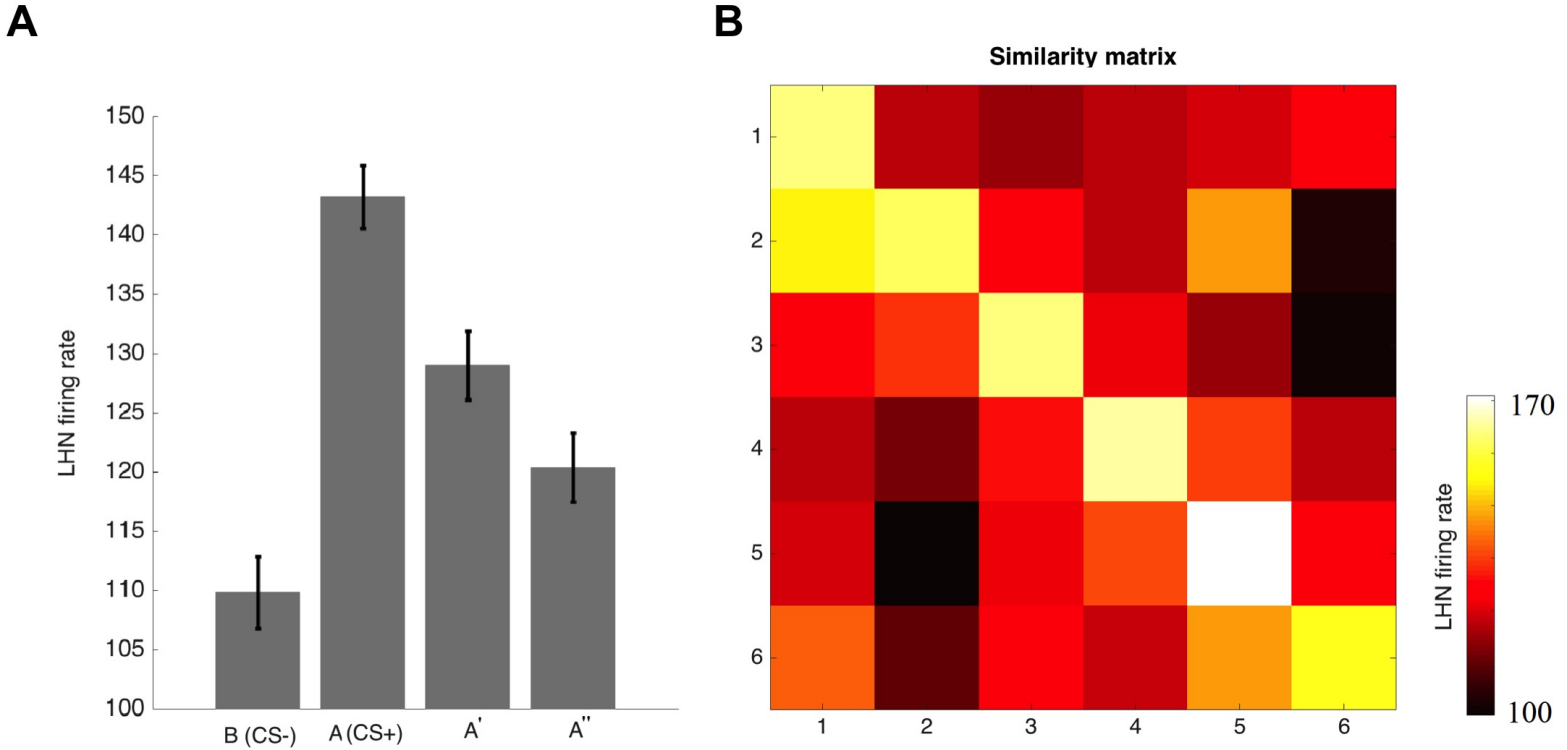
Olfactory generalization. A) The lateral horn neuron (LHN) responds to rewarded stimuli (CS) and two novel odorants with different level of the similarity to the CS (A’ is more similar to A than A”) after training to CS. LHN’s response to the A’ exhibits more perceptually similar to the CS for bees than to A”. B) The colour matrix shows the olfactory generalization matrix which represents the LHN response to six odours in the tests performed by bees trained with different CSs. Colour pixels (*i*,*j*) indicate the firing rate of LHN for the *j*th odour when the model was trained by the *i*th odours. The results show asymmetric generalization between odours.

To study whether our model can generalize from a learned odour to novel ones depending on odorant similarity, we conditioned the model following an absolute conditioning protocol, in which a single odorant (A) is paired with reward. The response of the lateral horn neuron for odour A reached a plateau before testing generalization. The model was then tested with two novel stimuli, one of which (A’) was similar to and the other (A”) different from the odour A. The distance of LHN responses in firing rate for the odour A and novel stimuli are assumed to represent the perceived similarity between A and other odours. The model responses in the tests following conditioning (Fig 6A) resemble the olfactory generalization performances found in honeybees [6,9], i.e. responses were higher for the odour A and decreased as a function of odour similarity. In order to evaluate the impact of odour similarity, we repeated our simulation but increased the number of test stimuli to six (the CS+ and 5 novel stimuli), with different levels of physical similarity. We defined physical similarity based on the Euclidean distance between vectors of 6 odour stimuli (see Method section). Fig 6B shows the similarity matrix, *K*, between the six odours. The colour of the element *K*_*i*,*j*_ denotes the firing rate of the lateral horn neuron for the *j*th stimulus when the model was trained to the *i*th stimulus. The performance of the model is consistent with experimental observations that showed asymmetrical generalization in honeybees [8]; for example, generalization from odour 3 to odour 5 is not the same as from 5 to 3. Because of the modulated plasticity within the antennal lobe during conditioning, the connectivity structure between glomeruli is re-shaped according to the activity of odour 3 while this structure is different if we train the model with another odour (odour 5) (S4 Fig). Hence, the activity within the antennal lobe and LHN for odour 3 when the model was trained with odour 5 is different from the activity of odour 3 when the model was trained by odour 5. Thus, an asymmetric similarity appears in the generalization matrix.

### Non-elemental olfactory learning

We next focused on the capacity of our network to solve non-elemental learning discriminations. We chose two types of non-elemental learning discrimination, which have been thoroughly investigated in honeybees, the positive and the negative patterning tasks [6,15,16]. In both tasks, bees have to discriminate a mixture odour AB from its components (A or B). In positive patterning, the odour components are non-rewarded and the compound is rewarded (A-, B-, AB+); in negative patterning, the components are rewarded and the mixture is not (A+, B+, AB-).

**Fig 7:**
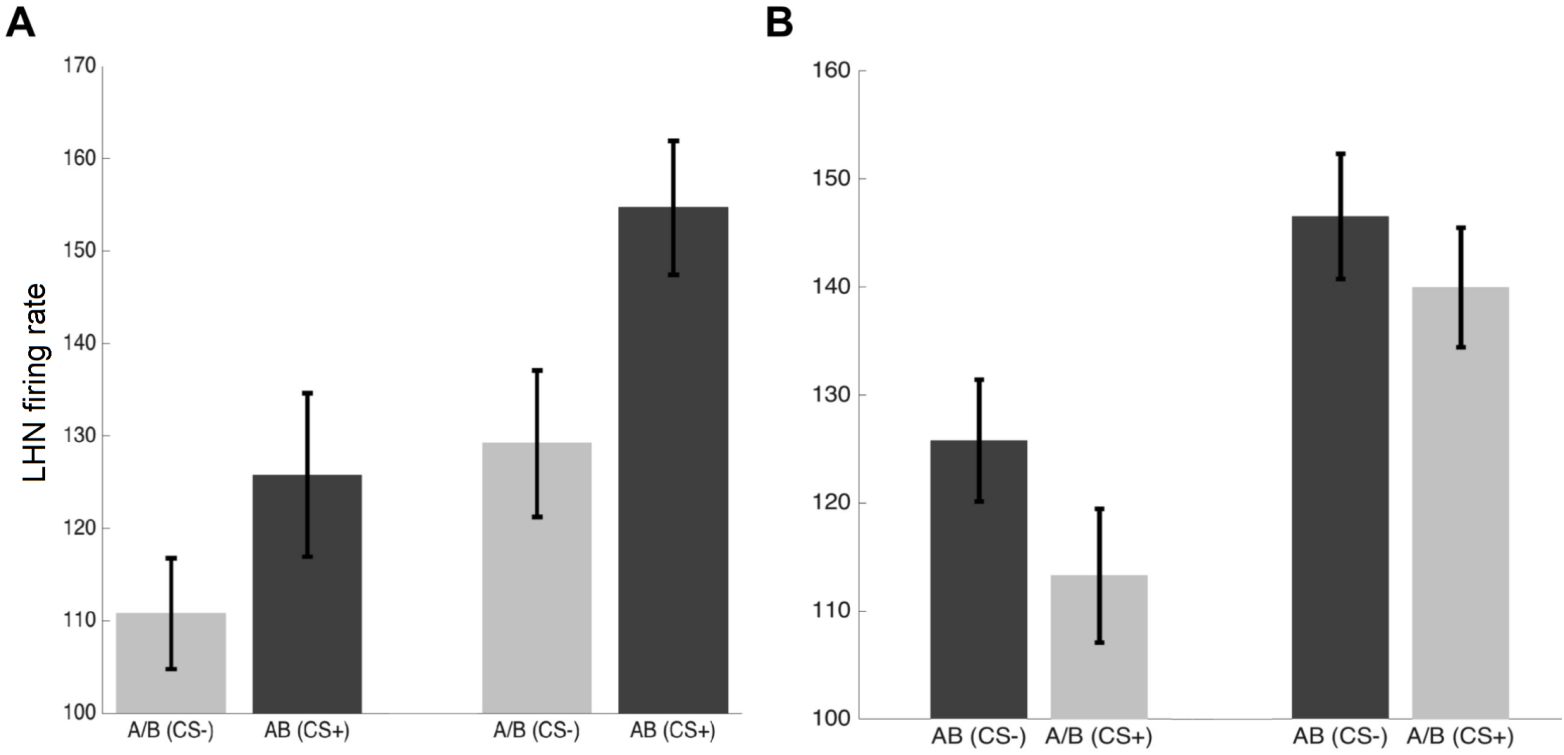
Non-elemental learning performance. A) Mean and SE of responses of LHN to unrewarded single odorants (A- or B-) and to a rewarded mixture odours (AB+) during the pre-training (left) and the test (right) of the conditioned PER. Simulated bees learned to discriminate mixture odorant AB from the single odorants A or B (n = 50, t-test; p-value < 0.003). B) Responses of LHN to rewarded mixture odorants (AB+) versus unrewarded components of the CS+ (n = 50, t-test; p-value = 0.23). The model was unable to learn the negative patterning tasks.

We first focused on positive patterning and paired the mixture odour AB with the reinforcement while components A or B were always unrewarded. Although the firing rate of lateral horn neuron for CS-s increased during training, the response of the lateral horn neuron to the CS+ (the rewarded compound AB) was significantly higher than that to the CS- (the components A/B; p-value = 0.003) (Fig 7A). This differentiation shows, therefore, that our model can achieve a positive patterning discrimination. Focusing on negative patterning yielded, however, a different result. In this case, the model was unable to differentiate between the unrewarded odour mixture AB and its components A and B (Fig 7B, p-value = 0.23). Hence, the model can solve positive but not negative patterning, confirming that both tasks differ in complexity [6] and might thus involve different neural circuits [15,35].

## Discussion

We introduced a spiking neural network model based on the l-ALT to evaluate the contribution of this particular tract of projection neurons to different forms of experience-dependent olfactory plasticity in honeybees. We first suggested a model of olfactory receptor neurons that provides realistic inputs to the antennal lobes (Fig 2). We then implemented non-associative plasticity rules between local neurons and projection neurons using iSTDP. We showed that the synaptic changes in the antennal lobe decrease correlations between projection neurons (Fig 3), thus enhancing olfactory differentiation at the output level of the antennal lobes (Fig 4). We then implemented octopamine–modulated plasticity in two synaptic regions of the olfactory circuit, the lateral horn and the antennal lobes in order to test for the role of such plasticity at the l-ALT level, i.e. upstream the mushroom bodies. In this way, we could focus on the capacity of our model to predict different classes of olfactory learning of variable complexity, i.e. on the contribution of the l-ALT to these different learning forms. We demonstrated that the model can achieve simple absolute conditioning and can generalize the learned olfactory information to novel stimuli depending on olfactory similarity with the conditioned stimulus (CS) (Fig 6). In addition, the model, with a few levels of processing, achieved differential conditioning and positive patterning discrimination (Figs 5 and 7). Yet, the model was unable to reproduce negative patterning (Fig 7B), thus showing that this non-elemental discrimination requires additional or separate circuitries (e.g. as provided by the mushroom bodies) and thus differs from the previous learning tasks. While recent modelling studies [5,19] suggested that m-ALT, which terminates in mushroom body extrinsic neurons, is sufficient for negative patterning, the mechanism of the negative patterning learning remains to be investigated.

### Non-associative plasticity in the antennal lobes

Young honeybees encounter a rich olfactory environment in the hive [55], which shapes their olfactory system. It has been shown that such passive olfactory exposure increases the volume of the honeybee brain, and also leads to structural modification [56]. Accordingly, odour exposures at early ages, in particular if associated with food reward obtained within the hive, modify sensitivity of the bees, influence performance in behavioural tasks, and make sensory representations in the antennal lobes significantly different from each other [57–59]. Thus, early olfactory experiences are likely to have a strong effect on the bee olfactory circuit in adult life. However, it is unclear which synapses in the antennal lobes are changing, leading to the observed bee behaviour. Galizia et al. [41] suggested that synapses between antennal lobe local neurons and projection neurons change their properties upon odour exposure. To test this hypothesis, we applied the iSTDP learning rule between inhibitory local neurons and excitatory projection neurons. We confirmed that the non-associative plasticity in the antennal lobes can change the random connectivity between glomeruli and create specific connectivity. Interestingly, the connectivity created by exposing the model to different odours increases the separability of odour representations at the antennal lobes output [24,32,33,43]. Moreover, the strength of the global inhibitory feedback neurons can regulate redundancy reduction and connectivity in the antennal lobes.

### Octopamine-induced plasticity mediates different forms of associative olfactory learning

Neural activity patterns at the level of the antennal lobes change shortly after learning, or after a long time after differential conditioning tasks [60–64]. More specifically, Rath et al. recorded calcium signals after differential conditioning and showed that two-odour response patterns in the antennal lobe for CS+ and CS- become more separable after a classical conditioning paradigm [60,61,63]. Further, the strength of calcium signals of the corresponding glomeruli increased in a conditioning task [61]. In particular, associative learning improved detectability of the corresponding glomeruli to an odour mixture from background activity [65]. However, the mechanisms underlying activity changes in antennal lobe activity after the conditioning task still remain elusive. Antennal lobe activity may be changed by internal sub-circuitry that were re-shaped via local plasticity rules within antennal lobes; alternatively it may be changed by feedback signals from the mushroom bodies [66–68]. Octopamine released from VUM-mx1 in the antennal lobes influences local neuron synapses [31,69,70]. Since octopamine is the reinforcement signal in the olfactory system of bees, learning-dependent activity in the antennal lobes might be caused by modulated plasticity between antennal lobe local neurons. In this study, we assumed this type of plasticity between local neurons, and showed a modification of neural representation in the antennal lobes as observed in experiments.

### Impact of plasticity between local neurons on olfactory generalization

After conditioning to the CS, a bee is able to respond to a novel stimulus whose perceived similarity is close to the CS (Fig 7A) [71,72]. It appears that bees generalize odours based on the similarity between carbon chain lengths or whether they belong to the same functional group [8]. Our study showed that generalization is not symmetric for several pairs of odours as asymmetric generalization was found for six odours that were randomly selected from the set of odours (Fig 6). The possible reason for obtaining such asymmetric structure in our model could be effect of the modulated plasticity between antennal lobe local neurons with the reward. S4 Fig shows how the initially random connectivity matrix between local neurons changes to specific connectivity, depending on the two different conditioned stimuli that activate different projection neurons within glomeruli. This causes different neural representation in the antennal lobes after training, and yields an asymmetric generalization [8].

### The mushroom bodies are dispensable for positive patterning but are required for negative patterning

Elemental and non-elemental learning are intimately related to classical classification problems. Theoretically, differential conditioning, positive, and negative patterning are equivalent to, respectively, OR, AND, and XOR problems in classification theory, with different levels of complexity. For instance, AND and OR problems can be solved by a single layer ‘perceptron’. It assigns different values to inputs of the network by discovering a linear plane [73]. However, single-layer perceptrons cannot solve the XOR problem because there are no planes that can be drawn across the space of inputs to separate the single components from their mixture. Numerous experimental studies have revealed better discrimination performance for positive patterning than for negative patterning [74–76]. However, a feed-forward network containing hidden units (multiple layers) can classify any inputs [77–78]. Thus, the medial pathway containing the Kenyon-cell layer connecting projection neurons and mushroom body extrinsic may allow bees to learn the negative patterning task [5,19].

Our model successfully solved the positive patterning task (A-, B- vs. AB+), but not the negative patterning discrimination (A+, B+ vs. AB-). This observation is interesting as it predicts that the former task could be solved just based on l-ALT circuitry, i.e. without mushroom body contribution. On the contrary, to solve a negative patterning task, the l-ALT circuitry would be insufficient and the downstream structure of the mushroom bodies would be required. This conclusion, however, contrasts partially with recent findings indicating that mushroom bodies are necessary both for positive and negative patterning discriminations [15]. Yet, these experiments relied on pharmacological blockade of mushroom bodies via procaine (or PTX in the case of PCT neurons), which supports the notion that these structures are necessary, but not sufficient for these forms of non-elemental learning. However, our model predicts that the activities of projection neurons in the case of stimulation with the single odours A, B and the mixture odour AB (as inputs of the decision neuron) are more separable when the network has been sufficiently modified by exposure to very different stimuli in the environment (Fig 4). Consequently, this modified neural network can solve the positive patterning task without participation of mushroom bodies through a linear classifier that also applies to the differential conditioning task. This indicates that adult honeybees, after extensive training, might be able to solve the positive patterning task even after blocking their mushroom bodies. This difference underlines the different associative nature of these two patterning problems: despite their apparent similar complexity (positive patterning may appear as a mirror-image discrimination with respect to negative patterning and vice versa), both tasks differ fundamentally in their difficulty. In fact, positive patterning discrimination could be solved through elemental learning because the associative strength of the non-rewarded components could be sub-threshold for the response but upon compound presentation they might result in a supra-threshold associative strength. Such a linear summation would yield higher associative strength and, therefore, higher responsiveness to the compound. This provides an elemental account of positive patterning, which is not possible in the case of negative patterning. Indeed, the negative patterning discrimination task can only be solved if the animal is able to process the mixture AB in non-linear terms. Otherwise, the sum of the excitatory strengths of the rewarded components upon compound presentation would always be greater than the strength of the single components. This difference may explain why fruit flies are able to master a positive patterning task but not negative patterning discrimination [18].

Fig 4B shows that the angular distance between glomeruli activated by the simultaneous presentation of odours A and B and those activated by the single odours A and B increases during non-associative learning. Hence, the number of antennal lobe neurons that fire for AB is greater than the number of neurons that fire for A and B. Some neurons fire selectively for the mixture AB but not for A or B. This should make synapses between these neurons (selective for AB) and the lateral horn neuron reinforced in positive patterning, leading to increased activity of LHN for the mixture AB. This result indicates that the spiking network acts as a linear classifier [15]. On the contrary, a specific neural circuitry has been recently identified as being necessary for negative pattern solving in honeybees [15]. PCT neurons which provide inhibitory GABAergic feedback to mushroom bodies are required for glomeruli in negative patterning [21,22]. These PCT neurons may reduce the activity of projection neurons in ventral region of the antennal lobe (i.e. inputs of m-ALT) for mixture odour AB. Therefore, the decision neuron in the next layer can discriminate odour components (A+, B+) from the mixture odour (AB-). In summary, our model predicts that, given appropriate experience of different odors in their early life, bees with lesions in the mushroom bodies may be able to solve the non-elemental positive patterning task but that the circuits outside the mushroom bodies are not sufficient for the negative patterning task.

### Assumptions of the model, and suggested experiments

There are substantial differences in the anatomy of the olfactory information processing system among different insect orders. Although the m-ALT is common to insects as diverse such as Orthopterans, Dipterans and Hymenoptera, the l-ALT is unique in the olfactory system of the latter [28]. Hence, it is possible that hymenopterans employ a different strategy for odour coding compared to other insects, thus enabling different olfactory learning abilities. Although it is important to understand the mechanism of the role of l-ALT as well as m-ALT in olfactory learning, our study focused on the l-ALT and its utility for olfactory learning. In modelling the l-ALT, we used only the anatomical and physiological evidence available for honeybees. One exception is the model of odour receptors, for which we used the neural properties of odour receptors of *Drosophila*, assuming that there are no significant differences between honeybees and fruit flies at the level of the peripheral odour encoding. Future studies must explore how the m-ALT and l-ALT interact during olfactory learning. Moreover, the learning performances of honeybees must be examined upon a specific lesion or blockade of the l-ALT, in particular this interface is performed at different places, i.e. before or after the lateral horn.

Many studies suggest that projection neurons might employ temporal coding for odour representation in antennal lobes where the temporal delay between odour onset and spiking activity of projection neurons might complement rate-based coding in the olfactory system [25,79–81]. We did not investigate such temporal coding in the proposed network because the available studies reported their results by *Ca*^2+^ imaging with low temporal resolution. Moreover, latency coding was observed mostly in the responses of projection neuron belonging to the m-ALT, and reports of latency coding in projection neurons of the l-ALT are rare [81–82]. This might indicate that temporal coding is not as prevalent in the l-ALT as in the m-ALT. However, studying the spatial and temporal coding in the dual olfactory system of honeybees will be an attractive topic for future studies. Note that our result suggests temporal information of spikes may be used by means of iSTDP for better odour separation in antennal lobes (Fig 4).

Our modelling predicts that non-associative learning changes the connectivity in the antennal lobes (Fig 3). Along with associative learning, our model further predicts that synaptic plasticity between local neurons and projection neurons in the antennal lobe may explain the individual difference in bee’s performance for the olfactory learning during their life (Fig 5). Behavioural and neurobiological investigations are needed to examine this prediction. We may compare learning performance of two groups of bees, one that explores different odours freely and another whose access to odours is limited in the early stages of their adult lives. We expect to find differences in the bees’ odour learning performance between the two groups in later life, and differently structured connectivity within the antennal lobes. Furthermore, one might discover distinct patterns of synaptic complexes within the antennal lobe for two groups of bees.

In a natural environment, bees can detect some odour plumes immediately [83–84]. However, the activity of projections neurons in the ventral regions of the antennal lobe is delayed relative those projection neurons in the l-ALT [25]. Moreover, mushroom body extrinsic neurons encode the value of the stimulus approximately 20 ms after the representation of odours in the lateral horn [81]. This evidence indicates that information transmission in the m-ALT is slower than the processing through the l-ALT. Thus, it could be more efficient for the olfactory system to recognise the identity of the odour stimuli by using rapid processing in the l-ALT. Moreover, concentrations of odour stimuli are evaluated by honeybees [38] although they are less important than identity coding. Hence, it could be proposed that m-ALT has a principal function in encoding odour concentrations. Schmuker et al. [32] suggested that strong lateral inhibition is useful for odour discrimination whereas gain-modulation by means of weak feedback inhibition is suitable for concentration discrimination. Moreover, it has been reported that responses of antennal lobe projection neurons in the l-ALT to weak odour concentrations are stronger than responses of antennal lobe projection neurons in the m-ALT [50]. Hence we expect to find stronger inhibition in the l-ALT as a gain control mechanism. Our results showed that strong inhibition in the dorsal region of the antennal lobe increased the performance of the model in odour discrimination through l-ALT. Further neurobiological studies are needed to investigate the impact of different levels of inhibition in dorsal and ventral regions of the antennal lobe for the coding of odour concentration and identity.

### Comparison with previous computational models

Computational models with different levels of complexities are critical to understand the bee olfactory system because a model can integrate biological evidence to link the function of neural networks to behaviour. Over the last decade, several models have been established to describe the characteristics of insect olfactory system from olfactory receptors to the mushroom bodies [5,19,34,44,85]. Many of the models focused on the role of antennal lobe networks in separating odour representations [32,44]. These studies showed that antennal lobe local neurons and their connectivity with projection neurons can improve performance of the classifiers that receive outputs from the antennal lobes [33]. A recent study also explored how synchrony between projection neurons can represent mixture odours differentially in the antennal lobes. Moreover, different coding strategies in the antennal lobe for odour identity and intensity were explained by the interaction of antennal lobe local neurons with a gain control neuron using a firing rate model [32,43]. Here, we reproduced similar results using a more realistic spiking neural network model, and additionally suggested how the specific connectivity between glomeruli emerges based on non-associative learning and different types of local neurons in the antennal lobe. Moreover, the current spiking-network model can be extended to investigate further questions in olfactory learning, for example, the effect of temporal separation between stimulus and reward presentation on learning performance [86–87].

Computational studies on the role of higher brain areas in insect cognition are scarce, and have mostly focused on mushroom bodies. For instance, Wessnitzer et al. developed a spiking model for *Drosophila* olfactory learning [19]. Their model followed Heisenberg’s approach [34], which considers the mushroom bodies as a main centre for associative olfactory learning. A recent study by using a binary network of the medial olfactory pathway examined the capacity of the mush room bodies in the different types of learning [5]. This study showed that reward-depending modification of synapses between Kenyon cells and the extrinsic output neurons of the mushroom bodies and the high sparseness of Kenyon cells allow the learning of complex discriminations such as the negative patterning, but the mushroom bodies are not necessary for elemental learning [15,35]. Hence, here we provided a minimal spiking neural network model of the l-ALT capable of reproducing some types of the learning without mushroom-boy requirement. A comparison between our model and others models of the medial olfactory pathway [5,19] suggests that an additional layer of processing with high sparse response might be essential for solving the negative patterning task.

## Methods

### Network topology

The model architecture of the honeybee lateral antennal lobe tract is shown in Fig 1B. Olfactory receptor neurons are activated by simulated odorant stimuli (see Odorant stimulus section) [30]. The olfactory receptor neurons then project to 36 glomeruli in the dorsal region of the antennal lobe, which is the primary site of olfactory processing in the l-ALT [28,35]. In each glomerulus, one projection neuron and one local neuron receive input from a single olfactory receptor neuron [88–89]. The glomeruli are laterally interconnected by the local neurons and projection neurons [90]. A local neuron in a glomerulus inhibits local neurons in the other glomeruli. The projection neuron in each glomerulus sends the excitatory signal to randomly selected local neurons in the other glomeruli. One global inhibitory neuron receives inputs from all projection neurons and sends a feedback signal to them. All projection neurons project to a neuron in the lateral horn called the lateral horn neuron (LHN) [29], which is the output of the present model. Finally, a VUM-mx1 neuron (shown in Fig 1B in yellow) makes reward-modulated connections with all the antennal lobe local neurons and the LHN [31]. We describe odorant stimuli and the function of each neuron in detail in the next subsections.

### Odorant stimulus

Odour molecules activate the initial stage of olfactory processing by producing nerve impulses in the olfactory receptor neurons. Odours contain a complex mixture of chemical compounds (i.e., ligands); therefore each odour is specified using a high-dimensional space of ligands [30,91]. An odour consists of a few ligands in this space of various concentrations. In this study, we present an odour in a vector of 36 elements *L* = (*l*_1_, *l*_2_,…, *l*_36_). Each element’s value exhibits the concentration of a particular ligand of the odorant. Because an odour typically contains 2 to 5 ligands [30], we randomly choose 2 to 5 elements, and assign the concentration values while unselected elements are fixed at zero (Fig 1C). The odour concentration ranges from minimum 10^−7^ to a maximum of 1 indicating the proportion of dilution. These patterns are used for the inputs to olfactory receptor neurons. This model captures some of the variability of the odour stimuli in the environment.

### Olfactory receptor neurons

Since the responses of olfactory receptor neurons (ORNs) are highly dynamic (i.e., their spike rates peak quickly and then relax to a tonic level of activity), we simulated responses pattern of ORNs to a large set of odorants by employing the adaptive exponential integrate-and-fire model (AdEx) [92]. By combining the AdEx model with the self-organizing model of receptors [90], we introduce a novel spiking neuron model that can generate the dynamic firing patterns of the olfactory receptor neurons. We constructed 360 olfactory receptor neurons composed of 36 different types (10 olfactory receptor neurons for each type). In this model, dynamics of sub-threshold membrane potential *v*_*i*_(*t*) of the *i*th olfactory receptor neuron (*i* = 1, …, 360) is described by the following two differential equations:

$$C^{ORN}\frac{dv_{i}(t)}{dt}=-g_{L}(v_{i}(t)-E_{L})+g_{L}\Delta_{T}exp(\frac{v_{i}(t)-V_{T}}{\Delta_{T}})-w_{i}(t)+I_{i}+\epsilon_{i}(t),$$ (1)

$$\tau_{w}\frac{dw_{i}(t)}{dt}=a(v_{i}(t)-E_{L})-w_{i}(t),$$ (2)

where *w*_*i*_(*t*) is an adaptation variable, *C*^*ORN*^ is membrane capacitance and *g*_*L*_ and *E*_*L*_ are leaked conductance and leak reversal potential, respectively. *Δ*_*T*_ (slope factor) is a time-scale of the adaptive threshold, and *a* (adaptation coupling parameter) and *τ*_*w*_ (adaptation time constant) are parameters for the adaptive membrane dynamics. The membrane potential *v*_*i*_ is reset to *v*_0_ if it exceeds the threshold, *V*_*T*_. Moreover, the adaptation variable, *w*_*i*_, is changed by an amount *b* (*w*_*i*_ → *w*_*i*_ + *b*). Here, the input to the model neuron is denoted as *I*_*i*_, which is computed from odour stimuli by using the self-organizing model of olfactory receptors. The details are described below. Finally, we added a Gaussian noise *ϵ*_*i*_(*t*) ∼ *N*(0, *σ*) to add randomness in the spiking activity of the olfactory receptor neurons. We set these parameters so that the model approximates characteristics of the olfactory receptor neurons [30,94] (See S1 Table for parameter values used in the simulation).

The input to the *i*th olfactory receptor neurons, *I*_*i*_, is calculated, following the model proposed in [93] as follows. First, the response *I*_*i*,*j*_ of the *i*th olfactory receptor neurons to ligand concentration, *l*_*j*_, of a stimulus *L* = (*l*_1_, *l*_2_,…, *l*_36_) is computed as:

$$I_{i,j}=\frac{1}{1+{\left(K_{i}^{j}l_{j}\right)}^{-m_{i}^{j}}},$$ (3)

where $K_{i}^{j}$ is the binding affinity of the *i*th olfactory receptor neurons to *l*_*j*_. The parameter $m_{i}^{j}$ denotes the molecular Hill equivalent, which represents a width of the effective concentration range encoded in the response of each olfactory receptor neuron with respect to the ligand *l*_*j*_. The input to the *i*th neuron, *I*_*i*_, is calculated as an average of the responses *I*_*i*,*j*_ to all ligands (*l*_*j*_) within the input stimuli *L*. The binding characteristic of the *i*th olfactory receptor neuron is thus specified by its affinity vectors, $K=(K_{i}^{1},\;K_{i}^{2},\ldots ,K_{i}^{36})$ and $M=(m_{i}^{1},\;m_{i}^{2},\ldots ,m_{i}^{36})$ (see S1 Fig). Here the vectors *K* and *M* exhibit the degree of sensitivity and selectivity of receptors to the stimulus. These vectors are generated randomly for 36 different types of olfactory receptor neurons as proposed in [19]. Although olfactory receptor neurons have high affinity for few ligands, most individual olfactory receptor neuron types respond to multiple ligands. The receptor responses saturate if concentrations of the active ligands are significantly high [30,95]. To realise the diverse selectivity and sensitivity of real olfactory receptor neurons, we assume that each olfactory receptor neuron exhibits a gradient of affinity to ligands while each olfactory receptor neuron possess a unique preferred ligand defined by the highest affinity.

### Antennal lobe model

In this study, we construct 36 types of olfactory receptor neurons (10 olfactory receptor neurons for each type, 360 in total) that converge onto 36 glomeruli in the antennal lobes. Further, we use a single projection neuron and antennal lobe local neuron for each glomerulus for simplicity. Since olfactory receptor neurons possessing similar response profiles to a ligand converge onto the same glomeruli, one projection neuron and one antennal lobe local neuron in each glomerulus receive an input from a single type of olfactory receptor neurons. This construction establishes the one-receptor for the one-glomerulus hypothesis for bee antennal lobes [89]. A local neuron in a glomerulus projects inhibitory connections to projection neurons in the other glomeruli. The same local neuron inhibits local neurons in the other glomeruli. We let the projection neuron in each glomerulus send weak excitatory signal to randomly selected local neurons in the other glomeruli (not shown in Fig 1) as is reported in [4]. Finally, the projection neurons in the antennal lobes send an excitatory signal to the higher-order centre of the brain. The activation of projection neurons causes global inhibitory feedback to themselves through a single global inhibitory neuron (GIN; a homogeneous local neuron) that receives inputs from all projection neurons.

In what follows, we explain in detail the spiking neuron models for these neurons. The subthreshold membrane potential of projection neurons ($u_{i}^{PN}$) and local neurons ($u_{i}^{LN}$) are described by the standard conductance-based leaky integrate-and-fire model. The membrane potential of projection neurons is given by:

$$\tau_{m}^{PN}\frac{du_{i}^{PN}(t)}{dt}=-u_{i}^{PN}(t)+R^{PN}I_{i}^{PN}(t),$$ (4)

where *R*^*PN*^ and $\tau_{m}^{PN}$ are resistance and membrane time constant of projection neurons respectively (see S2 Table for parameters). The input current $I_{i}^{PN}\;(t)$ represents synaptic inputs from olfactory receptor neurons, antennal lobe local neurons and a global inhibitory neuron as well as external noise. This input is written as

$$I_{i}^{PN}(t)=\sum_{j=1}^{N}\sum_{f}c_{i,j}^{ORN\to PN}\;g_{i}^{E}(t-t_{j}^{ORN})({V_{E}^{PN}-u}_{i}^{PN}(t))+\sum_{j=1}^{M}\sum_{f}c_{i,j}^{LN\to PN}\;g_{i}^{I}(t-t_{j}^{LN})({V_{I}^{PN}-u}_{i}^{PN}(t))+\sum_{f}c_{i,j}^{gLN\to PN}\;g_{i}^{I}(t-t^{f})({V_{gI}^{PN}-u}_{i}^{PN}(t))+I^{n}(t),$$ (5)

where *N* = 360 and *M* = 36 are the number olfactory receptor neurons and antennal lobe local neurons respectively. A positive scalar value $c_{i,j}^{ORN\to PN}$ specifies the strength of a synaptic input from the *j*th olfactory receptor neurons to the *i*th PN. Similarly, $c_{i,j}^{LN\to PN}$ and $c_{i,j}^{gLN\to PN}$ represent a synaptic weight of the *j*th LN to the *i*th PN, and a synaptic weight of GIN to PNs. Here we assume that each input spike ($t_{j}^{f}$; *f*: = olfactory receptor neuron, LN or GIN) cause conductance changes given by $g_{i}^{E}(t)=e^{-(t-t_{j}^{f})/\tau_{E}}\;(t\geq t_{j}^{f})$ for olfactory receptor neuron, and $g_{i}^{I}(t)=e^{-(t-t_{j}^{f})/\tau_{I}}\;(t\geq t_{j}^{f})$ for local neurons and GIN. We use synaptic time constants *τ*_*E*_ = 5*ms* for projection neurons and *τ*_*I*_ = 10*ms* for local neurons and *τ*_*I*_ = 20 *ms* for the GIN. To implement randomness in the activity in the antennal lobes, we add independent Gaussian noise *ϵ*_*i*_(*t*) ∼ *N*(0,*σ*) to the membrane potential of the projection neurons.

Similarly, local neurons are modelled by a conductance-based leaky integrate-and-fire model as:

$$\tau_{m}^{LN}\frac{du_{i}^{LN}(t)}{dt}=-u_{i}^{LN}(t)+R^{LN}I_{i}^{LN}(t),$$ (6)

where

$$I_{i}^{LN}(t)=\sum_{j=1}^{N}\sum_{f}c_{i,j}^{ORN\to LN}\;g_{i}^{E}(t-t_{j}^{ORN})({V_{E}^{LN}-u}_{i}^{LN}(t))+\sum_{j=1}^{M}\sum_{f}c_{i,j}^{PN\to LN}\;g_{i}^{E}(t-t_{j}^{PN})({V_{E}^{LN}-u}_{i}^{LN}(t))+\sum_{j=1}^{M}\sum_{f}c_{i,j}^{LN\to LN}\;g_{i}^{I}(t-t_{j}^{LN})({V_{E}^{LN}-u}_{i}^{LN}(t))+\epsilon_{i}(t)$$ (7)

Here, $c_{i,j}^{ORN\to LN}$ determines synaptic strength from the *j*th olfactory receptor neurons to the *i*th local neuron. We assume random sparse connectivity from projection neurons to local neurons $c_{i,j}^{PN\to LN}$ and LNs to LNs $c_{i,j}^{LN\to LN}$ (see S2 Table for parameters). The synaptic strengths from olfactory receptor neurons to local and projection neurons were adjusted so that the average activities of downstream local neurons to a stimulus become 40 Hz higher than the spontaneous spike rates [90]. In the subsequent method sections, we enrich the model by introducing non-associative and associative learning in the antennal lobe and lateral horn. There, the synaptic connections from local neurons to projection neurons are modified according to inhibitory spike-timing dependent plasticity (STDP) whereas the synaptic strength from LNs to LNs is modulated by octopamine during a learning procedure. We describe the details below.

### Non-associative learning in the antennal lobes

Recent studies revealed that non-associative plasticity modifies neural activities in the antennal lobes: neural representation of mixture odours is changed after bees are preferentially exposed to one component of the mixture without reward [95]. Further, the organization of the antennal lobes and honeybee's behavioural performances in learning tasks changes during the first week of their life apparently due to exposure to new stimuli [41]. The inhibitory synapses in the antennal lobes can be shaped by inhibitory spike-timing dependent plasticity (iSTDP) [96]. Here, we model non-associative learning by a symmetric iSTDP between presynaptic antennal lobe local neurons and postsynaptic projection neurons with a decay time constant *τ*_*iSTDP*_. In the symmetric iSTDP, both temporal ordering of pre- or postsynaptic spikes potentiate the connectivity, and the synaptic strength of *j*th inhibitory local neurons onto *i*th projection neurons ($c_{i,j}^{LN\to PN}$) is updated as follows. When we have a presynaptic event at time $t_{j}^{LN}$ of the j local neuron, the synaptic change is given by

$$\Delta c_{i,j}^{LN\to PN}=\eta (x_{i}^{PN}-\alpha ),$$ (8)

where $x_{i}^{PN}=\sum_{f}e^{-(t_{i,f}^{PN}-t_{j}^{LN})/\tau_{iSTDP}}$. Here, $t_{i,f}^{PN}$ exhibits the time of the *f*th postsynaptic spiking of *i*th projection neuron that appears before the presynaptic event ($t_{i,f}^{PN}<t_{j}^{LN}$). *η* is the learning rate. We added the depression factor *α* = 2 *ρ*_0_ *τ*_*iSTDP*_ (*ρ*_0_ is a constant) to control the target rate for the postsynaptic projection neuron [96]. When we have a postsynaptic event at $t_{i}^{PN}$ of the *i*th projection neuron, the synaptic change is given by

$$\Delta c_{i,j}^{LN\to PN}=\eta \;x_{j}^{LN},$$ (9)

where $x_{j}^{LN}=\sum_{f}e^{-\frac{t_{j,f}^{LN}-t_{i}^{PN}}{\tau_{iSTDP}}}$. Here, $t_{j,f}^{LN}$ exhibits the time of the *f*th presynaptic spiking of *j*th local neuron that appears before the postsynaptic event ($t_{j,f}^{LN}<t_{i}^{PN}$).

We assumed a random Gaussian connectivity matrix (Fig 2B) from LNs to PNs as an initial connectivity. This connectivity matrix is then modified according to the above procedure (Eqs 8 and 9) until it converges to stable synaptic strengths.

### Associative learning in the lateral horn

A population of projection neurons (l-PNs) transfer the olfactory signals to the lateral horn through the l-ATP. Further, the VUM-mx1 neuron releases octopamine in the antennal lobes, lateral horn and mushroom bodies [31]. Octopamine modulates synaptic changing for antennal lobe local neurons and the decision neuron (LHN) in the lateral horn [61,69], which is thought to underlie the reinforcement learning during appetitive conditioning. The spiking neuron model of LHN is described by the standard leaky integrate-and-fire model (see Eq 4 and S2 Table). In this study, we assume that all projection neurons convey their information to the single decision neuron (LHN) in the lateral horn. Synaptic strengths from the projection neurons to the LHN ($c_{1,j}^{PN\to LHN}$) are modified based on the STDP rule:

$$STDP(∆t)=\left\{ \begin{array}{c} A_{+}e^{-\frac{∆t}{\tau_{+}}}\;if\;∆t>0,\\ A_{-}e^{\frac{∆t}{\tau_{-}}}\;if\;∆t<0, \end{array}\right.$$ (10)

where Δ*t* = *t*_*post*_ − *t*_*pre*_ indicates the difference of spike times of presynaptic projection neuron (*t*_*pre*_) and postsynaptic LHN (*t*_*post*_). *A*_+_ and *τ*_+_ is the magnitude and time constant of the STDP function for synaptic potentiation whereas *A*_−_ and *τ*_−_ are constants for synaptic depression (see S3 Table for parameters). This STDP learning rule is modulated by octopamine release. The strengths of synapses are limited based on their capacity in changing. Here the effect of the octopamine is modelled as follows:

$$\Delta c_{1,j}^{PN\to LHN}=f_{d}(t)STDP(\Delta t),$$ (11)

where $f_{d}(t)=1\;if\;t<t_{reward},\;otherwise\;1+d\;e^{(t-t_{reward})/\tau_{d}}$ is the eligibility trace function which modulates the STDP function after the reward signal at time *t*_*reward*_. Here, *d* is the octopamine concentration and *τ*_*d*_, which increases or decreases the sensitivity of plasticity to delayed rewards. This equation is a simplified plasticity rule of modulated STDP suggested as a distal reward protocol [97].

## Supporting information

S1 Fig Parameters of the response curve of the olfactory receptor neuron model. A and B) left and right matrices show the molecular Hill equivalent and binding affinity for different olfactory receptor neuron (ORN) types, respectively. Index of ORNs is arranged in the vertical dimension. C) The probability distribution of the binding affinity for one ORN that represented in a row of the matrix B. Many of ORNs have high affinity for only a few ligands.
(TIF)

S2 Fig Examples of selectivity and sensitivity of olfactory receptor neurons. A) Firing rates of olfactory receptor neuron (ORNs) across 50 trials as a function of ligand concentration (solid line is mean, shaded area is standard error (SE)). The red curve indicates that this ORN is selective to Odour A. Here the slopes of curves show the degree of ORN sensitivity: A slope with greater value denotes higher sensitivity. B) The same presentation as in panel A for different ORN type. C) The Odour A and B used in the top panels are represented in two colour vectors.
(TIF)

S3 Fig Tuning response of 36 different olfactory receptor neuron types. A) Each histogram illustrates the firings rate of an olfactory receptor neuron (ORN) type activated by 100 random odours shown in panel B. The odours on the horizontal axis are sorted according to the firing rate while the higher firing rate is located closer to the centre. The red line indicates the spontaneous rate for each ORN. B) Rows in the coloured matrix represents 100 vectors of simulated odours.
(TIF)

S4 Fig Effect of associative learning in the dorsal region of the antennal lobe. Weight matrices of the synaptic connectivity between 36 local neurons (LNs) in the presence of modulated STDP are reformed in these connections (From left to right: random weights before training; weights after training to odour 3 and to odour 5). After conditioning, different response patterns were induced in the antennal lobes by odours 3 and 5.
(TIF)

S1 Table Parameters for firing patterns of olfactory receptor neurons. (DOCX)

S2 Table Parameters for firing patterns of projection neuron (PN), local neuron (LN) and lateral horn neuron (LHN). (DOCX)

S3 Table Parameters of the spike timing-dependent plasticity rule. (DOCX)

S1 Video Dynamic neural plasticity within the antennal lobe. The video shows how synaptic connectivity from 36 local neurons to 36 projection neurons within the antennal lobe varies with time. Thirty-six glomeruli are represented by nodes (arranged around a ring) whose numbers indicate the index of glomerulus in the model. Thickness of lines represents the strength of synaptic connectivity between local neurons and projection neurons. The initial connectivity values (first frame) were generated by a by a random Gaussian distribution. The connectivity between glomeruli are smoothly changed in presence of iSTDP between these connections by exposure to 1000 random odours shown on the right side of the video (see main text and Fig 3A).
(MP4)
