# Supplementary figures and images for: Olfactory learning without the mushroom bodies: Spiking neural network models of the honeybee lateral antennal lobe tract reveal its capacities in odour memory tasks of varied complexities
Source: PLoS Comput Biol. 2017 Jun 22;13(6):e1005551. doi: 10.1371/journal.pcbi.1005551 (PMC5480824; doi:10.1371/journal.pcbi.1005551)

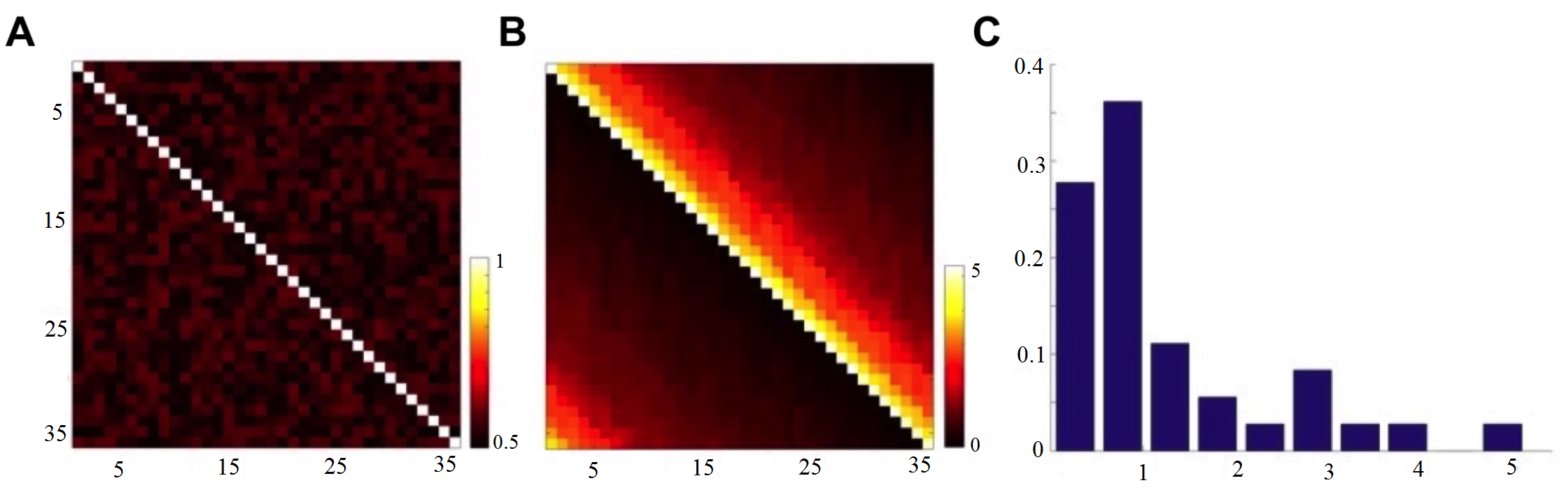

Supplement: S1 Fig — A and B) left and right matrices show the molecular Hill equivalent and binding affinity for different olfactory receptor neuron (ORN) types, respectively. Index of ORNs is arranged in the vertical dimension. C) The probability distribution of the binding affinity for one ORN that represented in a row of the matrix B. Many of ORNs have high affinity for only a few ligands. (TIF) [file pcbi.1005551.s001.tif]

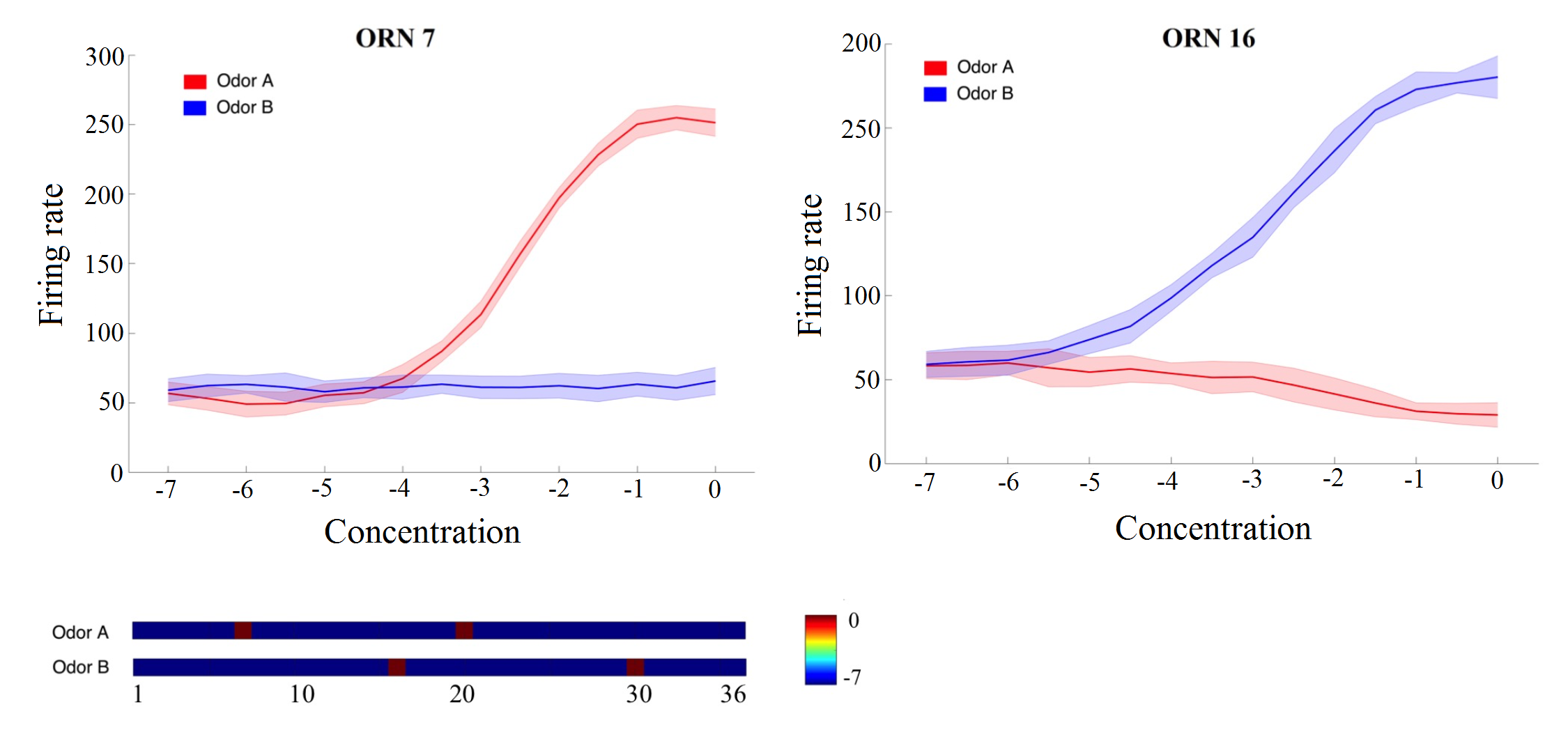

Supplement: S2 Fig — A) Firing rates of olfactory receptor neuron (ORNs) across 50 trials as a function of ligand concentration (solid line is mean, shaded area is standard error (SE)). The red curve indicates that this ORN is selective to Odour A. Here the slopes of curves show the degree of ORN sensitivity: A slope with greater value denotes higher sensitivity. B) The same presentation as in panel A for different ORN type. C) The Odour A and B used in the top panels are represented in two colour vectors. (TIF) [file pcbi.1005551.s002.tif]

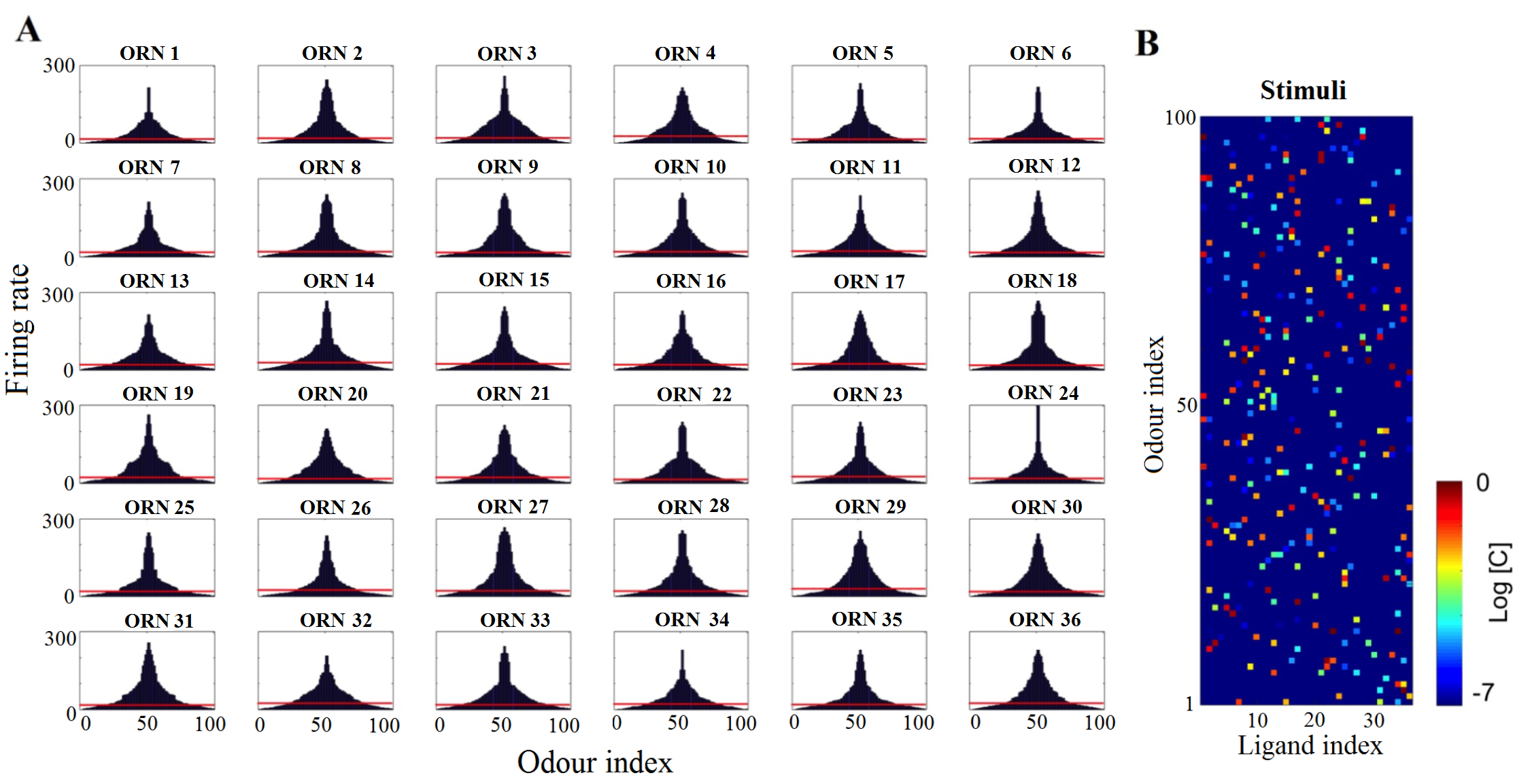

Supplement: S3 Fig — A) Each histogram illustrates the firings rate of an olfactory receptor neuron (ORN) type activated by 100 random odours shown in panel B. The odours on the horizontal axis are sorted according to the firing rate while the higher firing rate is located closer to the centre. The red line indicates the spontaneous rate for each ORN. B) Rows in the coloured matrix represents 100 vectors of simulated odours. (TIF) [file pcbi.1005551.s003.tif]

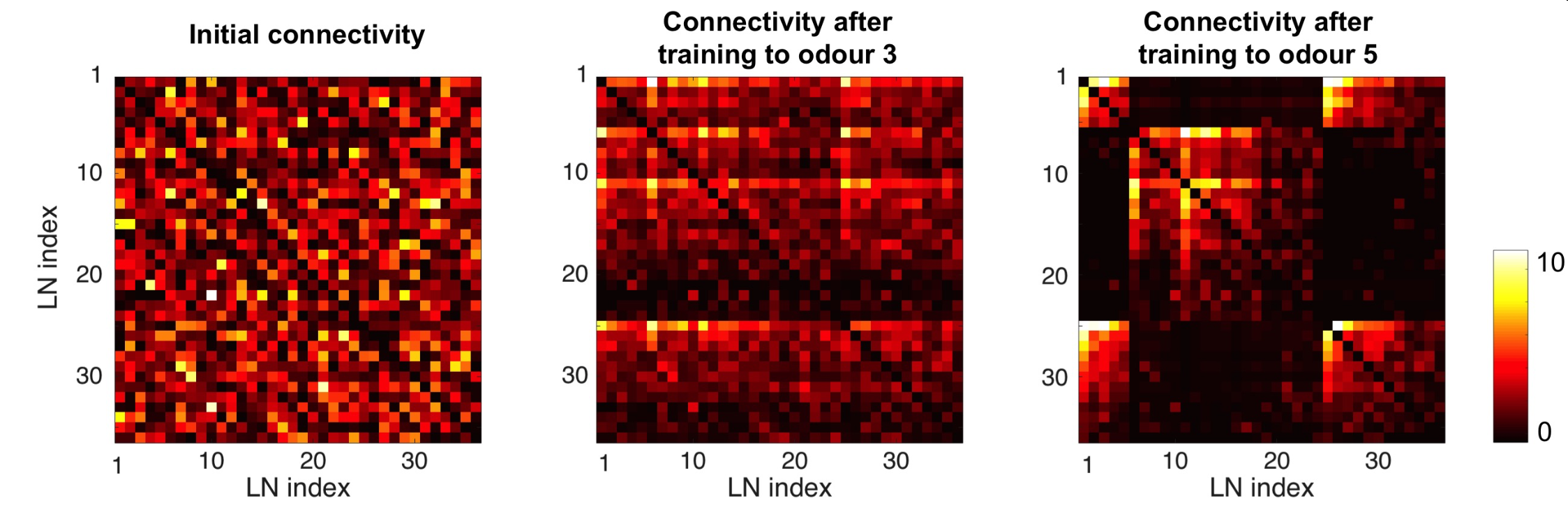

Supplement: S4 Fig — Weight matrices of the synaptic connectivity between 36 local neurons (LNs) in the presence of modulated STDP are reformed in these connections (From left to right: random weights before training; weights after training to odour 3 and to odour 5). After conditioning, different response patterns were induced in the antennal lobes by odours 3 and 5. (TIF) [file pcbi.1005551.s004.tif]
