# Supplementary material for: Olfactory learning without the mushroom bodies: Spiking neural network models of the honeybee lateral antennal lobe tract reveal its capacities in odour memory tasks of varied complexities
Source: PLoS Comput Biol. 2017 Jun 22;13(6):e1005551. doi: 10.1371/journal.pcbi.1005551 (PMC5480824; doi:10.1371/journal.pcbi.1005551)
Supplement: S1 Table — (DOCX) [file pcbi.1005551.s005.docx]

**S1 Table.** Parameters for firing pattern of olfactory receptor neurons.

|  | C  (pF) | $g_{L}$ (nS) | $E_{L}$ (mV) | $V_{T}$ (mV) | $\Delta_{T}$ (mV | a  (nS) | b  (pA) | $V_{r}$ (mV) | $\tau_{w}$  (mS) |
| --- | --- | --- | --- | --- | --- | --- | --- | --- | --- |
| ORN | 200 | 12 | -70 | -50 | 2 | 2 | 60 | -58 | 300 |
