# Supplementary material for: Olfactory learning without the mushroom bodies: Spiking neural network models of the honeybee lateral antennal lobe tract reveal its capacities in odour memory tasks of varied complexities
Source: PLoS Comput Biol. 2017 Jun 22;13(6):e1005551. doi: 10.1371/journal.pcbi.1005551 (PMC5480824; doi:10.1371/journal.pcbi.1005551)
Supplement: S2 Table — (DOCX) [file pcbi.1005551.s006.docx]

**S2 Table.** Parameters for firing pattern of projection (PN), local neuron (LN) and lateral horn neuron (LHN).

|  | $\tau_{m}$  (mS) | R  (m$\Omega$) | $V_{E}$  $(mV)$ | $V_{I}$  (mV) |
| --- | --- | --- | --- | --- |
| PN, LN, LHN | 10 | 10 | 0 | -80 |
