# Supplementary material for: Olfactory learning without the mushroom bodies: Spiking neural network models of the honeybee lateral antennal lobe tract reveal its capacities in odour memory tasks of varied complexities
Source: PLoS Comput Biol. 2017 Jun 22;13(6):e1005551. doi: 10.1371/journal.pcbi.1005551 (PMC5480824; doi:10.1371/journal.pcbi.1005551)
Supplement: S3 Table — (DOCX) [file pcbi.1005551.s007.docx]

**S3 Table.** Parameters of the spike timing-dependent plasticity rule.

| $A_{+}$ | $\tau_{+}$  (mS) | $A_{-}$ | $\tau_{-}$  (mS) |
| --- | --- | --- | --- |
| 10 | 20 | 10 | 20 |
